# Comparison of ELISA Versus FAMA Titers in Children After Chemotherapy and Hematopoietic Stem Cell Transplantation Who Received the Live Attenuated MAV/06 Strain Varicella Vaccine

**DOI:** 10.3390/vaccines12121371

**Published:** 2024-12-05

**Authors:** Bin Ahn, Kyu Ri Kang, Ye Ji Kim, Yoon Kyung Cho, Suejung Jo, Jae won Yoo, Jae Wook Lee, Nack-Gyun Chung, Bin Cho, Dae Chul Jeong, Jin Han Kang, Hyun Mi Kang

**Affiliations:** 1 Vaccine Bio Research Institute, College of Medicine, Catholic University of Korea, Seoul 06591, Republic of Koreakch323@naver.com (K.R.K.); yeji882@catholic.ac.kr (Y.J.K.);; 2 Department of Pediatrics, College of Medicine, Catholic University of Korea, Seoul 06591, Republic of Koreahoiring0209@gmail.com (J.w.Y.); dashwood@catholic.ac.kr (J.W.L.); cngped@catholic.ac.kr (N.-G.C.);

**Keywords:** hematopoietic stem cell transplantation, immunogenicity, MAV/06, varicella zoster virus

## Abstract

Background: Varicella can lead to severe complications in immunocompromised children, including those undergoing hematopoietic stem cell transplantation (HSCT) or chemotherapy. Preventing primary varicella zoster virus (VZV) infection is crucial in these populations to mitigate morbidity and mortality. This study aimed to evaluate the immunogenicity and safety of the live attenuated MAV/06 varicella vaccine in pediatric patients post-HSCT and post-chemotherapy. Additionally, it sought to compare fluorescent-antibody-to-membrane-antigen (FAMA) and enzyme-linked immunosorbent assay (ELISA) titers to establish effective cut-off levels for protection against varicella. Methods: The FAMA assay was conducted at the Vaccine Bio Research Institute, and a VARICELLA-ZOSTER ELISA (Vircell, Granada, Spain) kit, which relies on lysate from whole cells infected with VZV, was used to determine VZV IgG. A prospective cohort study was conducted with 76 pediatric patients under 18 years old who tested negative for VZV IgG via ELISA. Patients post-HSCT and post-chemotherapy were included. Participants received the MAV/06 varicella vaccine, and serologic responses were evaluated using ELISA and FAMA. Results: The median age of participants was 9.8 years, with acute lymphoid leukemia and acute myeloid leukemia being the most common underlying disease. Post-dose 1, the seropositive rate was 56.1% by ELISA and 97.2% by FAMA. Based on the FAMA seropositive cut-off ≥1:4, post-dose 1 geometric mean titers (GMTs) of seropositive patients in the post-HSCT group were 14.7 (95% CI, 11.3–19.1) versus 20.2 (95% CI, 13.0–31.3) in the post-chemotherapy group (*p* = 0.690). Based on a FAMA seropositive cut-off ≥1:16, the post-dose 1 GMT of patients considered seropositive in the post-HSCT group was 19.3 (95% CI, 15.6–24.0) versus 34.1 (95% CI, 21.0–55.4) in the post-chemotherapy group (*p* = 0.116), and post-dose 2 FAMA titers of 76.1 (95% CI, 14.6–398.1) in the post-HSCT group and 64.0 (95% CI, 11.4–358.1) in the post-HSCT group (*p* = 0.853) were observed. In patients with lower baseline FAMA titers (1:4 to 1:8), 66.7% in the post-HSCT group and 71.5% in the post-chemotherapy group achieved a greater than four-fold increase in FAMA titers post-dose 1, while those with higher baseline titers (≥1:16) did not. There were no serious adverse events or vaccine-related rashes occurring in any of the patients. Conclusion: The MAV/06 varicella vaccine is immunogenic in pediatric patients post-HSCT and post-chemotherapy, particularly when administered in a two-dose schedule using a cut-off FAMA titer of <1:16.

## 1. Introduction

Varicella zoster virus (VZV) infections, while typically mild in healthy children, can lead to severe complications such as secondary bacterial infections, pneumonia, and encephalitis, which can require hospitalization or result in fatal outcomes [1]. The risk of severe VZV infections is significantly higher in immunocompromised children, including those undergoing chemotherapy, organ transplant recipients, and individuals with Human Immunodeficiency Virus infections, leading to considerable morbidity and mortality. [2,3,4]. Research on immune reconstitution in children following chemotherapy reveals that although total B-cell numbers recover rapidly, an imbalance between memory and naïve B cells persists, and disruptions in the T-cell compartment can still be observed at 18 months [5]. In post-hematopoietic stem cell transplantation (HSCT) patients, B-cell recovery is further delayed, with incomplete immune restoration within the first two years after allogeneic transplantation [6]. These findings highlight the lasting impact of both chemotherapy and HSCT on immune function, affecting both susceptibility to infections such as varicella, and responses to immunization.

Primary VZV infection results in the virus becoming latent in the sensory nerve ganglia, with the potential to reactivate later in life as herpes zoster (shingles), a condition that typically affects the elderly. Preventing the initial varicella infection can significantly reduce the risk of developing shingles, which can be a painful and debilitating condition [7]. Reactivation is particularly common following HSCT, and breakthrough infections or late reactivations occur even with antiviral prophylaxis [8,9,10]. Therefore, preventing primary infections in this group of the population is extremely important.

Vaccines designed to prevent primary varicella infections include various live attenuated varicella strains, such as vOka-based strains, MAV/06-based strains, and Oka/SK strains. While numerous studies have demonstrated the immunogenicity of these vaccines in healthy children, there are limited data on vulnerable populations such as pediatric allogeneic (allo)-HSCT recipients. One study on children that received the Oka strain varicella vaccine post-HSCT reported that 58.1% (18 out of 31) were seropositive after vaccination, reflecting a relatively low response rate [11]. Another study found a 64% seroconversion rate after one dose of the varicella vaccine post-HSCT in T-replete or T-cell-depleted alloHSCT patients [12]. Both studies assessed responses using enzyme immunoassays. Furthermore, as varicella vaccines are live vaccines with reported adverse events in both immunocompetent and immunocompromised individuals by different viral strains [13,,14,15], careful monitoring of its safety in these patients is essential. However, current data on this matter remains insufficient.

The fluorescent-antibody-to-membrane-antigen (FAMA) test is considered gold standard for detecting protective antibodies against VZV [16]. However, it is labor-intensive and requires specialized equipment for handling the virus and ensuring protection against exposure [17]. An enzyme-linked immunosorbent assay (ELISA) offers a less labor-intensive alternative for measuring VZV antibodies. Despite this convenience, commercial ELISAs are less sensitive than the traditional FAMA assay, and protective antibody cut-off levels for varicella differ between the two methods and are not yet clearly established. Unfortunately, most hospitals in South Korea rely solely on the ELISA method to assess and interpret VZV IgG serostatus in children.

The FAMA titer of the serum sample is determined by the highest dilution that still results in a positive ring-like fluorescence reaction. Research indicates that healthy children with serum FAMA titers of ≥1:4 for VZV are generally protected from chickenpox infection [18]. However, FAMA has a lower performance in immunocompromised patients, and studies have shown that even if FAMA indicates the presence of detectable antibodies to VZV, immunocompromised patients still develop mild varicella upon exposure [19]. As for glycoprotein ELISA (gpELISA), several breakthrough infections have been reported in healthy children despite them having glycoprotein ELISA (gpELISA) titers of ≥5 EU/mL. This raises the question of whether a higher cut-off titer may be necessary to ensure adequate protection. According to the 2006 European Sero-Epidemiology Network 2 (ESEN2) guideline, a conventional ELISA result of <50 mIU/mL is considered seronegative [20]. One study demonstrated that when a cut-off value of <50 mIU/mL is used, the FAMA titer of 1:16 achieves optimal sensitivity, specificity, and correlation. This finding suggests that the current FAMA cut-off level for varicella protection, particularly after vaccination, should be re-evaluated [21], at least for vulnerable populations.

Therefore, the objective of this study was to evaluate the immunogenicity and safety of the live attenuated MAV/06 varicella vaccine in pediatric patients who underwent allogeneic HSCT (allo-HSCT) and are candidates for re-immunization, and to compare these results with those of children who received chemotherapy and are candidates for catch-up vaccination. Additionally, since the ELISA method is the only option available for assessing VZV IgG serostatus in children at most hospitals, this study aimed to investigate the correlation between FAMA and ELISA titers in this population and to establish cut-off levels that may enhance protection against varicella infections.

## 2. Materials and Methods

### 2.1. Study Participants

This prospective cohort study included patients under 18 years of age who tested negative for VZV IgG via the ELISA method and were not receiving immunosuppressive agents or immunomodulators. The inclusion criteria for the post-HSCT group were: (1) an absolute CD4+ T-cell count of at least 200 cells/mm³ and (2) at least two years post-HSCT. For the post-chemotherapy group, patients were eligible if at least six months had passed since their last chemotherapy treatment. This study was approved by the Institutional Review Board (IRB) of Seoul St. Mary’s Hospital, The Catholic University of Korea (IRB no. KC22SISI0491).

### 2.2. Study Design

At our pediatric bone marrow transplant center, all patients who have undergone HSCT or completed chemotherapy are required to have their VZV serostatus evaluated as part of the standard protocol. Similar to most hospitals, only the ELISA method is available for VZV IgG testing. As such, patients were screened using ELISA to determine their baseline VZV IgG levels. Those with an ELISA index of <9, indicating seronegativity, were identified as candidates for varicella vaccination and enrolled in the study after obtaining informed consent. Remnant blood samples were stored for FAMA analysis. Patients were then immunized with the live attenuated MAV/06 strain Barycela vaccine. Four to twelve weeks after the first dose, a post-dose 1 blood sample was taken to assess the serologic response using ELISA, with remnant samples stored for FAMA analysis. Patients with negative post-dose 1 VZV IgG (ELISA) results received a second dose of the Barycela vaccine. Four to twelve weeks after the second dose, a post-dose 2 blood sample was taken for serologic testing using ELISA, with remnant samples again stored for FAMA analysis. At the end of the study period, all pre-dose 1, post-dose 1, and post-dose 2 blood samples were analyzed using FAMA, and VZV IgG (ELISA) and FAMA titers were compared.

### 2.3. Vaccination Protocol

The live attenuated varicella vaccine immunization protocol for post-HSCT patients at our hospital during the study period adhered to the 2020 National Immunization Program recommendations, the IDSA guidelines, and the ACIP guidelines for immunocompromised hosts [16,17,18]. Patients under 13 years of age received a single dose of the live attenuated varicella vaccine, followed by serologic testing using VZV IgG (ELISA). If the test result was negative, a second dose was administered. Patients aged 13 years and older received a series of two doses. In post-chemotherapy patients, VZV IgG was screened six months after the final chemotherapy session, and one or two doses were administered based on the vaccination history prior to initiating chemotherapy.

For this study, the majority of patients were evaluated for serologic response after both the first and second doses. Patients with sampling failures, those who did not consent to additional testing, or those without available samples had missing post-dose 1 or 2 FAMA or ELISA titers. During the study period, patients received the varicella vaccine based on VZV IgG determined by the ELISA method, which is the standard protocol at our center, since FAMA results were not immediately available.

### 2.4. Immunogenicity Evaluation Methods

Anti-Varicella zoster immunoglobulin WHO International Standard W1044 (50 IU/vial) was purchased from NIBSC as the standard antiserum for varicella. MRC-5 cells were obtained from the European Collection of Authenticated Cell Cultures (ECACC) (Cat No. 05072101), specifically the 19th passage (pd19) with Cat No. 05072101. The MAV/06 strain was provided by GC Biopharma (Yongin-si, Republic of Korea). The VARICELLA-ZOSTER ELISA kit (Vircell, Granada, Spain) (Lot 21EVZV102), which relies on lysate from whole cells infected with VZV, was used to determine VZV IgG using the ELISA method. Results are expressed as an index, with >11 indicating a positive result and <9 indicating a negative result, as recommended by the manufacturers.

The FAMA assay was conducted at the Vaccine Bio Research Institute, College of Medicine, The Catholic University of Korea, Seoul, Korea, using a modified version of previous methods. MRC-5 cells were cultured in DMEM medium containing 10% fetal bovine serum, 100 U/mL penicillin, 100 µg/mL streptomycin sulfate, and 1% L-glutamine for 7 days. The cells were then infected with the MAV/06 strain in DMEM medium containing 25 mM HEPES and 2% fetal bovine serum, and cultured for an additional 4–5 days. After confirming the cell morphology, the cells were seeded onto 10-well slides at a concentration of 5–10 × 10^5^ cells/mL, with 25 µL per well, incubated overnight in a humidity chamber, dried at 45 °C, and stored frozen until use. Each well was treated with 20 µL of fixation buffer, blocked with a solution containing 0.1 M glycine, 0.4% rabbit serum, and 0.05% Tween 20 in PBS for 5 min, and incubated with serum samples diluted from 1:2 at 30 µL per well for 2 h. After incubation, FITC-conjugated anti-human antibody (Dako, Hamburg, Germany) diluted 1:50 was added at 25 µL per well, incubated for 1 h, and nucleic acids were stained with DAPI at 300 nM concentration (1 µL in 30 mL). The results were observed using a Carl Zeiss Axiovert 200 Fluorescence Microscope (Oberkochen, Germany). Serum and secondary antibody dilutions were prepared using PBS, and intermediate washing steps were performed twice for 2 min each with PBS + 0.05% Tween 20. The slides were dried thoroughly before proceeding. The dilution factor at which more than 50% of cells formed a FITC-stained membrane was recorded as the titer, with titers ≥ 1:4 considered positive. Serial dilutions were performed up to 1:512 for analysis. The results were validated by two independent observers. FAMA titers were used to assess the serologic response. In this study, a FAMA titer of ≥1:4 was considered positive. The reciprocals of the FAMA titers (i.e., 1:4 = 4, 1:16 = 16) were used to determine the geometric mean titer (GMT).

### 2.5. Study Definitions

Seropositive was defined as follows: for FAMA, >50% membrane staining at ≥1:4 dilutions were considered positive, and for ELISA (Vircell kit), an index >11 was considered positive. Seroconversion was defined for FAMA as a conversion from <1:4 to ≥1:4 dilutions after immunization. For ELISA, IgG from index <9 to ≥11 after immunization was considered seroconversion. Patients with titers 9–10 were considered equivocal, and were considered seronegative in the seroconversion analyses.

### 2.6. Statistical Analyses

The seroconversion rate was calculated as the number of seroconverted patients divided by the total number of patients tested multiplied by 100, with a 95% confidence interval (CI) using the Clopper–Pearson method. The seroconversion rate for both the FAMA and ELISA methods was calculated based on patients who were identified as seronegative by each respective method at baseline. The seropositivity rate was calculated as the number of seropositive patients divided by the total number of patients tested multiplied by 100. The geometric mean ratio (GMR) was calculated as post-vaccination GMT divided by pre-vaccination GMT. The Wilcoxon signed-rank test was used to compare GMTs of a group pre- and post-vaccination, and the Mann–Whitney U test for comparing groups. Data were analyzed using MS Office Excel and PrismTM software v9.02 (GraphPad, La Jolla, CA, USA), with the results expressed on a logarithmic scale using an analysis of variance model.

## 3. Results

### 3.1. Study Population

**Figure 1:**
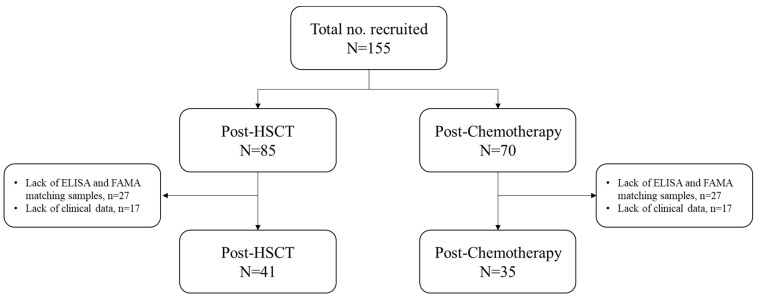
Flow chart of the patients included in this study. A total of 42 patients in the post-hematopoietic stem cell transplantation and 35 in the post-chemotherapy groups were included. ELISA, enzyme-linked immunosorbent assay; FAMA, fluorescent-antibody-to-membrane-antigen; HSCT, hematopoietic stem cell transplantation.

A total of 76 participants were enrolled in this study (Figure 1), and the median age of the patients was 9.8 years old (interquartile range [IQR], 7.3–13.9). Acute lymphoid leukemia (ALL) was the most common underlying disease (61.8%, n = 47/76), followed by acute myeloid leukemia (AML) (19.7%, n = 15) and severe aplastic anemia (SAA) (6.6%, n = 5) (Table 1).

By groups, the median age of the patients was 11.0 (IQR, 8.2–14.2) years old in the post-HSCT group, and 8.6 (IQR, 7.1–11.2) years old in the post-chemotherapy group (*p* = 0.090). The interval from HSCT to dose 1 of the varicella vaccine was 2.4 (IQR, 2.1–3.1) years in the post-HSCT group, whereas, the interval from the last chemotherapy to dose 1 of the varicella vaccine was 1.1 (IQR, 1.1–1.3) years (*p* < 0.001). In both the post-HSCT and post-chemotherapy groups, the most common underlying disease was ALL (46.3% vs. 80.0%) followed by AML (26.8% vs. 11.4%) (*p* = 0.017), respectively. The majority of the patients in the post-HSCT group received unrelated allogeneic HSCT (36.6%, n = 15) followed by familial mismatched HSCT (26.8%, n = 11). The median CD4+ T cell count of the patients was 634.4 (IQR, 450.6–1096.2) per 10^9^/L, and the median CD19+ B cell count was 651.2 (351.5–872.7) per 10^9^/L.

### 3.2. Seropositive Rate, Seroconversion Rate, and Sensitivity of ELISA

**Figure 2:**
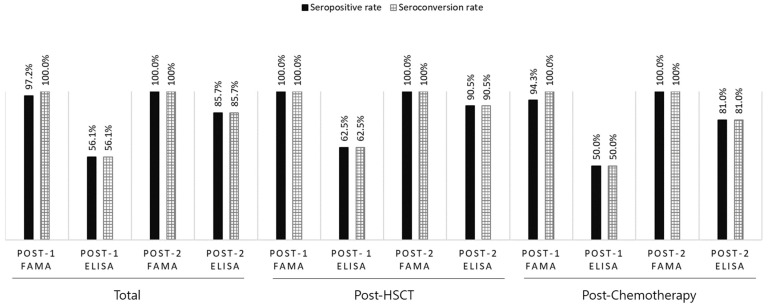
Comparison between FAMA and ELISA to assess seropositive and seroconversion rates of the patients included in this study. The vertical axis represents the rate in percentages. ELISA, enzyme-linked immunosorbent assay; FAMA, fluorescent-antibody-to-membrane-antigen; HSCT, hematopoietic stem cell transplantation.

Because seronegative patients were enrolled based on ELISA VZV IgG < 9, the post-dose 1 seropositive rate and seroconversion rates were the same when assessing immunogenicity by ELISA. Overall seropositive and seroconversion rates of 56.1% (n = 37/66) post-dose 1 and 85.7% (n = 36/42) post-dose 2 via ELISA were observed. However, using FAMA, the seropositive rate was 97.2% (n = 70/72) post-dose 1 and 100% (n = 23/23) post-dose 2. The seroconversion rate was 100% post-dose 1 and 2 (Figure 2). The seropositive and seroconversion rates by group are shown in Figure 2. Based on a FAMA cut-off titer of 1:4, the sensitivity of post-dose 1 ELISA VZV IgG was 59.0% and the specificity was 100%.

### 3.3. Geometric Mean Titers and Ratios by Groups

Although all patients were seronegative for VZV IgG based on ELISA, based on the FAMA seropositive cut-off titer ≥1:4, 31.7% (n = 13/41) of the patients in the post-HSCT group and 25.7% (n = 9/35) in the chemotherapy group were found to be seropositive prior to vaccination. Based on the FAMA seropositive cut-off ≥1:4 and ≥1:8, the post-dose 1 GMT of seropositive patients in the post-HSCT group was 14.7 (95% CI, 11.3–19.1) versus 20.2 (95% CI, 13.0–31.3) in the post-chemotherapy group, showing no significant difference (*p* = 0.690).

**Figure 3:**
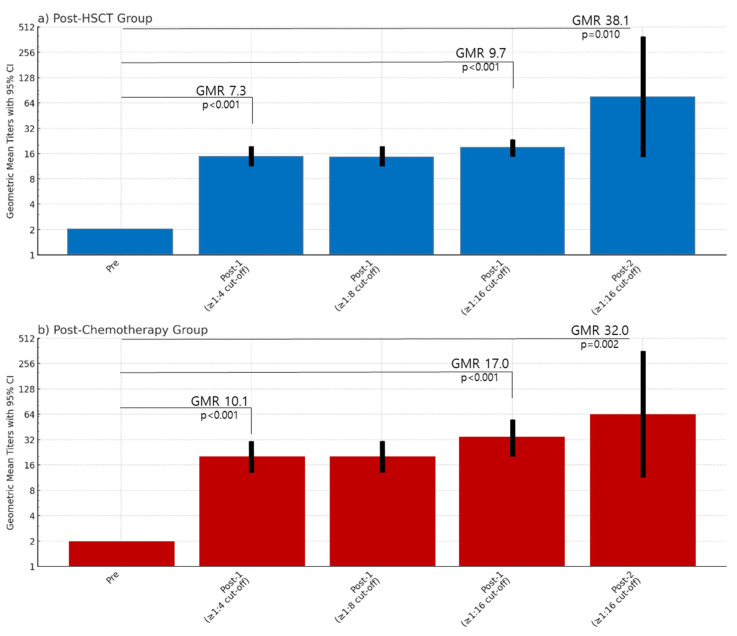
The geometric mean titers and ratios based on 1:4, 1:8, and 1:16 seropositive cut-off references in the post-hematopoietic stem cell transplantation and post-chemotherapy groups. GMR, geometric mean ratio; HSCT, hematopoietic stem cell transplantation.

In an analysis of GMTs based on a FAMA seropositive cut-off ≥1:16, the post-dose 1 GMT of patients considered seropositive in the post-HSCT group was 19.3 (95% CI, 15.6–24.0) versus 34.1 (95% CI, 21.0–55.4) in the post-chemotherapy group, showing no significant difference (*p* = 0.116). Furthermore, using this cut-off of ≥1:16, patients considered seronegative (FAMA < 1:16) that were given a second dose of varicella vaccine had post-dose 2 FAMA titers of 76.1 (95% CI, 14.6–398.1) in the post-HSCT group and 64.0 (95% CI, 11.4–358.1) in the post-HSCT group, without a significant difference (*p* = 0.853) (Table 2). In both the post-HSCT group and chemotherapy groups, compared to the pre-vaccination FAMA titers, the GMR increased significantly (Figure 3).

### 3.4. Titer Fold Increase Depending on Pre-Vaccination Baseline FAMA Titer

**Figure 4:**
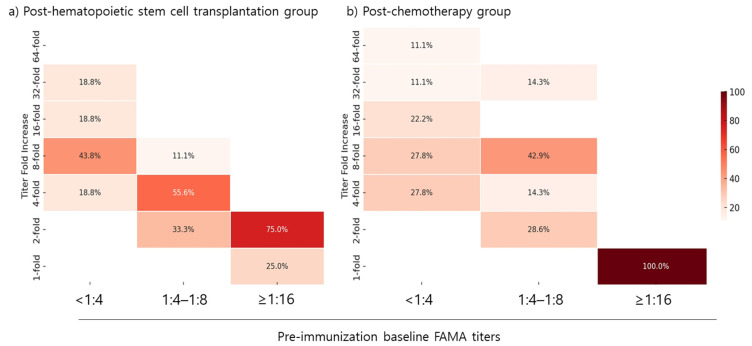
Titer fold increase depending on pre-vaccination baseline FAMA titer. FAMA, fluorescent-antibody-to-membrane-antigen; HSCT, hematopoietic stem cell transplantation.

The fold increase in FAMA titers following the first vaccination dose was evaluated based on patients’ baseline FAMA titers. In both the post-HSCT and post-chemotherapy groups, all patients with a pre-vaccination baseline FAMA titer of <1:4 achieved a greater than four-fold increase in FAMA titers. Among patients with a baseline FAMA titer of 1:4–1:8, 66.7% in the post-HSCT group and 71.5% in the post-chemotherapy group achieved a greater than four-fold increase in FAMA titers post-dose 1. However, none of the patients with a baseline FAMA titer of ≥1:16 in either group achieved a greater than four-fold increase (Figure 4).

### 3.5. Adverse Events Monitoring

For all patients, adverse events were monitored actively for up to 42 days. Injection site swelling, erythema, and pain were observed up to 3 days post-vaccination in 2.6% (n = 2/76) of the patients, one in the post-HSCT group and one in the post-chemotherapy group. All patients were monitored passively. However, no varicella-like rash or herpes-zoster-like lesions were observed in any of the patients. A fever up to 38.0 °C was observed in 1.3% (n = 1/76) of the patients, and this patient was in the post-chemotherapy group. There were no immune-mediated symptoms, including thrombocytopenia, anaphylaxis, or erythema multiforme and no neurologic syndromes such as neuropathy, convulsion, or encephalopathy.

## 4. Discussion

This study evaluated the immunogenicity and safety of the live attenuated MAV/06 varicella vaccine in pediatric patients post-HSCT and post-chemotherapy. Our study enrolled 76 pediatric participants, with a median age of 9.8 years, primarily diagnosed with ALL and AML. The seropositive and seroconversion rates post-vaccination were assessed using ELISA and FAMA methods. Post-dose 1, the seropositive rate was 56.1% by ELISA and 97.2% by FAMA. Both post-HSCT and post-chemotherapy groups exhibited significant increases in GMTs after vaccination, with the post-dose 2 GMTs aligning with those observed in healthy children [22]. The fold increase in FAMA titers was notably higher in patients with lower baseline titers (<1:4) but minimal in those with baseline titers ≥1:16, suggesting that initial antibody levels influence vaccine response.

HSCT requires the removal of existing bone marrow through conditioning, which also eliminates existing immune cells, antibodies, and memory T-cells from prior vaccinations, necessitating re-vaccination [23]. Post-HSCT patients who are seronegative for VZV antibodies and have CD4 counts above 200 cells/µL are recommended to receive the live varicella vaccine two years post-transplant [24,25]. In contrast, patients who completed chemotherapy and are in remission only require catch-up vaccination. In this study, 100% of participants seroconverted after the first MAV/06 varicella vaccine dose (FAMA-based). Furthermore, post-dose 1 GMTs indicated no significant difference between the post-HSCT and post-chemotherapy groups (14.7 vs. 20.2, *p* = 0.690), reinforcing the vaccine’s consistent immunogenicity across different patient cohorts. Nevertheless, the GMTs in this group of patients were lower than those observed post-dose 1 in the phase 3 clinical trial of this vaccine, which included healthy children with a GMT of 74.2 (95% CI, 65.0–84.8) [22]. Post-dose 2 GMTs in our study were found to be comparable to those of healthy children, and the substantial increase in GMT post-dose 2 for FAMA-negative patients further validates the booster dose’s role in enhancing immunogenicity. These findings support the completion of a two-dose schedule in patients post-HSCT and post-chemotherapy.

Although FAMA and gpELISA are highly sensitive immunologic assessment tools for varicella, they are frequently unavailable commercially in Korea. Consequently, the ELISA method is widely used. However, the low correlation between ELISA and FAMA is a known issue [17]. Otani et al. reported the following sensitivity and specificity for different ELISA methods: gpELISA (67% and 100%), EIA (67% and 100%), and IAHA (47% and 100%) [26]. The sensitivity observed in our study was similarly low at 59.0%, aligning with another study conducted on Korean children, which showed a VZV IgG seropositive rate of 83.6% via FAMA but only 44.8% via ELISA [27]. While a FAMA titer of <1:4 is generally used as the reference for seronegativity, the 2006 European Sero-Epidemiology Network 2 (ESEN2) guidelines define a conventional gpELISA result of <50 mIU/mL as seronegative [20]. This gpELISA threshold has been shown to correlate best with a FAMA titer of 1:16 [21]. Moreover, a study using a FAMA titer of 1:16 as a cut-off for seropositivity in healthy children still showed a 20.1% rate of breakthrough varicella [28]. This indicates that higher FAMA titers may be necessary for maximum protection and that a positive FAMA result does not necessarily equate to long-term immunity. Therefore, if we were to use a FAMA titer of <1:16 as the reference for seronegativity in post-HSCT or post-chemotherapy patients, the GMTs for patients considered seropositive would be higher, and a greater number of patients would be candidates for a second dose of the varicella vaccine.

Despite initial ELISA-based seronegativity before vaccination, a notable proportion of patients were found seropositive by FAMA. This observation suggests a potential residual immunity or cross-reactive antibodies undetected by ELISA, and also portrays the low sensitivity of ELISA for the detection of VZV IgG in patients. Due to their lower sensitivity, ELISA results may lead to patients who are seropositive by FAMA receiving an unnecessary dose of the varicella vaccine, as observed in our study. Therefore, it is logical to assess the benefits of vaccination in these patients. Our study found that the majority of patients with a seropositive FAMA titer between 1:4 and 1:8 benefited from the vaccine, demonstrating a four-fold or greater increase in FAMA titers. However, patients with a seropositive FAMA titer of 1:16 showed a limited response, with minimal or no increase in titers after vaccination. This outcome shows that initial antibody levels influence the subsequent vaccine response in post-HSCT and post-chemotherapy patients, indicating a need for tailored vaccination strategies. Furthermore, based on these findings, a FAMA titer of <1:16 may be an appropriate cut-off level, especially in high-risk populations that may benefit from increased serum titers.

This study had several limitations. The relatively small sample size and single-center design may limit the generalizability of our findings. However, data on the response to varicella vaccines in this high-risk pediatric population are scarce. Additionally, the reference for absolute protection remains unclear with both FAMA and ELISA. Therefore, reliance on serologic markers as proxies for clinical protection necessitates further investigation through longitudinal studies assessing actual varicella infection rates post-vaccination. Finally, the correlation analysis between FAMA and ELISA was not included because it was limited to patients who were VZV IgG-negative by ELISA at baseline.

## 5. Conclusions

To conclude, our findings reveal that the FAMA assay, despite its complexity, remains the gold standard for detecting VZV IgG antibodies, highlighting the limitations of the more commonly used ELISA method in clinical settings. The significant difference in seropositive rates between ELISA and FAMA underscores the higher sensitivity of FAMA. While ELISA indicated a lower seropositive rate post-vaccination, FAMA demonstrated a near-complete seroconversion, particularly after the second dose. These results demonstrate the necessity of a two-dose varicella vaccination schedule in immunocompromised pediatric patients to achieve optimal immunogenicity. The study also emphasizes the importance of considering baseline antibody levels when administering the varicella vaccine. Patients with lower baseline FAMA titers (1:4 to 1:8) showed a substantial increase in antibody levels post-vaccination, whereas those with higher baseline titers (≥1:16) exhibited limited response. Therefore, a FAMA titer of <1:16 may serve as a more appropriate reference for determining the need for a second vaccine dose, especially in high-risk populations. Finally, no significant adverse events or vaccine-related rashes were observed after immunization with this live-attenuated vaccine.

## Figures and Tables

**Table 1:** Demographics of the patients included in this study.

	Total n = 76	Post-HSCT n = 41	Post-Chemotherapy n = 35	*p*
Male sex, no. (%)	35 (46.1)	17 (41.5)	18 (51.4)	0.524
Age, median years (IQR)	9.8 (7.3–13.9)	11.0 (8.2–14.2)	8.6 (7.1–11.2)	0.090
Interval from end of treatment to dose-1, median years (IQR)	2.1 (1.1–2.6)	2.4 (2.1–3.1)	1.1 (1.1–1.3)	<0.001
Underlying disease, no (%)				
ALL	47 (61.8)	19 (46.3)	28 (80.0)	0.017
AML	15 (19.7)	11 (26.8)	4 (11.4)	
SAA	5 (6.6)	5 (12.2)	-	
HLH	2 (2.6)	2 (4.9)	-	
JMML	2 (2.6)	2 (4.9)	-	
Lymphoma	2 (2.6)	-	2 (5.7)	
CML	1 (1.3)	-	1 (2.9)	
PID *	2 (2.6)	2 (4.9)	-	
HSCT type				
unrelated		15 (36.6)		
FMM		11 (26.8)		
MSD		8 (19.5)		
cord blood		7 (17.1)		
CD4+ T cell count, median, 10^9^/L (IQR)		634.4 (450.6–1096.2)		
CD19+ B cell count, median, 10^9^/L (IQR)		651.2 (351.5–872.7)		

Abbreviations; ALL, acute lymphoid leukemia; AML, acute myeloid leukemia; CML, chronic myeloid leukemia; HLH, hemophagocytic lymphohistiocytosis; JMML, juvenile myelomonocytic leukemia; PID, primary immunodeficiency; SAA, severe aplastic anemia. * PID includes SCID (n = 1) and Blackfan-Diamond (n = 1).

**Table 2:** Seroconversion, geometric mean titer, and geometric mean titer ratio of patients in the post-HSCT and post-chemotherapy groups by FAMA.

Cut-Off Titer		≥1:4				≥1:8		≥1:16					
Parameters	Pre	HSCT Post-1	Chemo Post-1	*p*	HSCT Post-1	Chemo Post-1	*p*	HSCT Post-1	Chemo Post-1	*p*	HSCT Post-2	Chemo Post-2	*p*
Seropositive rate, %		100	94.3		100	94.3		81.1	68.6		100	100	
Seroconversion rate, %		100	100		100	100		68.8	66.7		100	100	
(95% CI)								(46.04–91.46)	(44.89–88.44)				
GMT	2.0	14.7	20.2	0.690	14.7	20.2	0.690	19.3	34.1	0.116	76.1	64.0	0.853
(95% CI)	(2.0–2.0)	(11.3–19.1)	(13.0–31.3)		(11.3–19.1)	(13.0–31.3)		(15.6–24.0)	(21.0–55.4)		(14.6–398.1)	(11.4–358.1)	
GMR		7.3	10.1		7.3	10.1		9.7	17.0		38.1	32.0	
(95% CI)		(5.7–9.5)	(6.6–15.5)		(5.7–9.5)	(6.6–15.5)		(7.8–12.0)	(10.8–27.0)		(11.1–130.1)	(13.1–78.1)	

Seroconversion—no. of patients included in the HSCT group, n = 16; post-chemotherapy group, n = 18. GMT for vaccines that seroconverted. Based on seropositive cut-off level 1:16, patients that were administered a second dose in the HSCT group, n = 4; chemotherapy group, n = 3. Abbreviations: chemo, chemotherapy; GMR, geometric mean ratio; GMT, geometric mean titer; HSCT, hematopoietic stem cell transplantation.

## Data Availability

Partial data is available upon request to the corresponding author.
